# Standards and Ontologies for Functional Genomics 2

**DOI:** 10.1002/cfg.448

**Published:** 2004-12

**Authors:** Midori A. Harris, Helen Parkinson

**Affiliations:** European Bioinformatics Institute EMBL Outstation Wellcome Trust Genome Campus Hinxton Cambridge CB10 1SD UK


**Comparative and Functional Genomics**
*Comp Funct Genom* 2004; **5**: 618–622.
Published online in Wiley InterScience (www.interscience.wiley.com). **DOI:** 10.1002/cfg.448

Conference Editorial

### Standards and Ontologies for Functional Genomics 2

University of Pennsylvania, Philadelphia, PA, USA, 23–26 October 2004

Midori A. Harris* and Helen Parkinson
*European Bioinformatics Institute, EMBL Outstation, Wellcome Trust Genome Campus, Hinxton, Cambridge CB10 1SD, UK*

**Correspondence to:*
*Midori A. Harris, EMBL-EBI- The European Bioinformatics Institute, Wellcome Trust Genome Campus, Hinxton, Cambridge CB10 1SD, UK.*
*E-mail: midori@ebi.ac.uk*

Received: 26 November 2004
Accepted: 26 November 2004

Like the first conference, held in 2002, on Standards and Ontologies for Functional Genomics (**www.sofg.org**), the second SOFG meeting brought together computer scientists, ontologists and biomedical scientists in Philadelphia to examine the state of the art in ontology technology, development and emerging standards for biomedicine.

Briefly, an ontology is a means of formalizing knowledge about a subject; at a minimum, an ontology must include terms (concepts) relevant to a domain, definitions for the terms, and defined relationships between the terms. Ontologies may be represented in any of a number of formats and systems, with varying degrees of complexity, and may range in size from hundreds to hundreds of thousands of concepts. In biomedicine, ontologies support the organization and management of large amounts of data, permitting sophisticated searches and allowing database annotations to be standardized. The standards aspect of the conference focused on content standards, i.e. what information should be captured about a biological concept or an experiment. This issue of 'what should be said' leads naturally to the consideration of how things can be said, thus forging connections between standards and ontologies. Format was touched upon in the context of representing and exchanging biological data, experimental metadata (data about data) and ontologies themselves.

SOFG2 examined the progress of the world of ontology and standards development in the two years since SOFG1; a number of significant changes soon became evident.

Notably, the ontology community has adopted the I3C standard Web Ontology Language (OWL; Horrocks *et al.*, 2003), the successor to DAML + OIL (Stevens *et al.*, 2002), as its lingua franca. In parallel with the move to OWL, the past two years have brought increasingly sophisticated tools for building and applying ontologies to the community. Presentations and discussions at SOFG2, summarized briefly below, identified several more common issues and themes.

In her keynote presentation, **Carole Goble** used Shakespeare's *Romeo & Juliet* as an analogy to illustrate the sociological (we currently know of no romantic) interactions between ontology developers coming from differing perspectives. As described in detail in the accompanying review (Goble and Wroe, 2004), computer scientists and philosophers (the 'Montagues') emphasize formal structures, whereas domain experts such as

Copyright © 2005 John Wiley & Sons, Ltd.

biologists (the ‘Capulets’) have preferred less formal, more pragmatic approaches, despite the limitations inherent in informal systems. The Montague/Capulet analogy struck a chord with participants, as several speakers declared their allegiance; notably, many proudly claimed connections with both formalist and pragmatist camps. Although the relationship between the formal and pragmatic camps has historically been marked by tension and conflict, an important theme emerging from SOFG2 is that of growing mutual interest and respect, as computer scientists become attracted to the complex use cases that biology provides, and biologists come to appreciate the practical benefits of formal ontological approaches. The final outcome may thus be happier for bio-ontologists than for Shakespeare’s lovers, thanks to the efforts of our ‘Princes of Genomics’ who straddle both communities, and perhaps also to the auspicious location of SOFG2 in the city of brotherly love.

**Chris Wroe** began the first session, on ***Ontological Systems: Theory and Development***, with a presentation that followed logically from Goble’s, giving an overview of both theoretical and practical aspects of ontology development. For example, an ontology may be represented by a simple structure such as a hierarchy or directed acyclic graph (DAG), or in a sophisticated formal structure such as a description logic (DL) system. The most suitable representation depends on the requirements, i.e. on the intended use of the ontology. An ontology may be converted from a simpler to a more formal representation as needs evolve, as illustrated by the example of the GONG project (Wroe *et al.*, 2003), which represented a portion of the Gene Ontology (GO) in OWL.

Two presentations then described uses of OWL to adapt ontologies for particular purposes: **Chintan O. Patel** discussed motivations for, and challenges presented by, representing existing biomedical domain ontologies in OWL; **Karim Nashar** presented a proposal to integrate several ontologies, including the MGED core ontology (Stoeckert and Parkinson, 2003) and existing and proposed extensions, to represent experiment metadata. To complete the session and link with the following session, **Michael Ashburner** summarized the origins and aims of the Open Biology Ontologies (OBO; formerly GOBO) initiative (**obo.sourceforge.net**). Intended to extend the model of community-based development of publicly available ontologies begun with GO, OBO has grown to encompass over 40 ontologies, covering diverse biological domains. The availability of certain ontologies, such as those for anatomical terms or chemical substances, facilitates the creation and maintenance of combinatorial concepts. Many types of biological knowledge can only be adequately represented by such compound concepts; an example particularly relevant to SOFG is that of modelling phenotypes, which requires concepts from anatomy, experimental procedures (assays) and results, among others. GO and OBO have also given rise to the development of OBOL, a language for formalizing combinatorial terms in OBO ontologies.

The session on ***Ontologies for Biological Systems*** began with two perspectives on biological pathways. The Reactome database (**www.reactome.org**), presented by **Peter D’Eustachio**, models the entities and events that make up pathways. **Minoru Kanehisa** described the graph-based design of KEGG (Kanehisa *et al.*, 2004). In light of comments made in several talks on the need for an ontology of chemicals, **Marcus Ennis** gave a very timely presentation on the nascent database of Chemical Entities of Biological Interest (ChEBI) (**www.ebi.ac.uk/chebi**). ChEBI covers chemical nomenclature, formulae, and structures, and is built upon a chemical ontology that organizes chemical compounds by both chemical characteristics and function, essentially providing a biologist’s view of the chemical world. The last talk tied the session to the preceding one: **Chris Catton** used a description of the BioImage database (**www.bioimage.org**) and its ontology-based architecture to provide context for a discussion of challenges that ontology developers face, especially in a field such as biology, where knowledge changes rapidly and sometimes substantially.

The third session, ***Ontology Systems Development: Electronic Demonstrations***, underscored the considerable progress that has been made since SOFG1 in developing tools to construct ontologies and applying them in biological contexts. **Mark Musen** and **Phil Lord** presented different aspects of Protégé (Noy *et al.*, 2003): Musen gave an overview of the tool, noting recent developments such as support for multiple simultaneous users, and a growing array of plugins that lend support for OWL reasoning and ontology management. Lord then focused on the OWL plugin, which allows Protégé to read and write OWL ontologies,

Copyright © 2005 John Wiley & Sons, Ltd. 

and provides an interface for constructing description logic statements. Users who have worked with DL statements in less user-friendly editors will welcome these advances. **John Day-Richter** reported on recent enhancements in DAG-Edit (**godatabase.org/dev/index.html**), which was originally created to edit GO and other DAG-structured ontologies, and now supports many features of more sophisticated representations. DAG-Edit now offers a powerful mechanism for searching, filtering and displaying ontology terms, and a wide array of plugins to support managing categories, parents and namespaces. Another plugin allows DAG-Edit to use OBOL; still others track change history and provide a graphical display. **Barry Zeeberg** demonstrated GoMiner (Feng *et al*., 2003; Zeeberg *et al*., 2003), one of several tools that combines GO data with gene expression data to aid interpretation of large-scale data in an ontology-based context. These tools and others were then included in an 'electronic poster' session, in which individual tool demonstrations occurred.

The session on ***Thesauri, Nomenclatures, and the Biomedical Literature*** highlighted several aspects of working with free text as well as ontologies. As noted by **Stuart Nelson**, the Unified Medical Language System (UMLS; McCray and Nelson, 1995) is not an ontology, but addresses some of the same issues as biological ontologies. UMLS integrates information from disparate sources based on conceptual connections that aim to resolve ambiguities in usage. **Inderjeet Mani** then described the PRONTO protein ontology, produced by a tool that automates much of the information gathering process. Another approach to mining literature and other data sources came from **Winston Hide**'s talk on applying the eVOC anatomy ontology to interpret expression data (Hide *et al*., 2003). **Yves Lussier** discussed the need to map between different ontologies, as well as incorporate data from many different genomic datasets, to deal with phenotype data or the 'phenome' on a large scale (Lussier and Li, 2004).

***The Standards and Protocols*** session began with two talks on anatomy ontologies and how they are used to integrate anatomy with biological information at different scales and in different experimental contexts. **John Gennari** described the Foundational Model of Anatomy (Rosse and Mejino, 2003), with its focus on human anatomy, formal structure, and precise definitions. Notably, the FMA includes the subcellular anatomical structures also covered by the GO cellular component ontology; some inconsistencies between the GO cellular component ontology and the FMA have come to light, and should be resolved in the near future. The Anatomical Dictionary for the Adult Mouse (**www.informatics.jax.org/searches/anatdict_form.shtml**), presented by **Terry Hayamizu**, has a somewhat simpler structure than the FMA, and is used by the mouse community to annotate their data. The anatomy talks continued a theme that was among the highlights of SOFG1, where discussions began that led to the creation of the SOFG Anatomy Entry List (Parkinson *et al*., 2004), a simple high-level anatomy mapping ontology. Work continues to map between anatomy ontologies.

**Duncan Davidson** returned to the recurring problem of modelling phenotypes. Two important issues facing databases are: (a) sharing and searching phenotype data across species; and (b) mapping between formally decomposed representations and shorthand phrases familiar to biologists. A simple system using characters, attributes and values (CAV) has been proposed and shows promise. These issues were revisited in a breakout session devoted to the Phenotype and Trait Ontology (PATO) (**obo.sourceforge.net/cgi-bin/detail.cgi?poav**), an ontology of attributes and values designed for phenotype representation. Annotations using PATO would refer to other ontologies, such as GO, anatomy ontologies, developmental stage ontologies and so on, for the characters in a CAV system.

**Chris Stoeckert** reviewed the MGED ontology (Stoeckert and Parkinson, 2003) for microarray experiment description, noting its relationship to the MAGE object model and to external ontologies. In the near future the ontology must be extended to accommodate new technologies and biological areas of interest. Both points were considered further in a breakout session focused on the extension of the MGED ontology into the areas of functional genomics, proteomics, environmental biology and toxicogenomics, as well as the need to support the emerging MAGE2 model, which will move beyond microarrays into functional genomics.

The success of the MGED ontology and the ubiquitous MIAME standard for microarray data (Brazma *et al*., 2001) has inspired analogous efforts for other experiments and data types, as illustrated by **Eric Deutsch**'s talk on the MISFISHIE standard

Copyright © 2005 John Wiley & Sons, Ltd. 

(**scgap.systemsbiology.net/data/misfishie/**; named after a bathtub toy) for gene expression localization experiments, and **Michael Cary**'s presentation on BIOPAX (**www.biopax.org**), an exchange format for pathway data. Developed for use by some 138 pathway databases, BioPax uses the high-level concepts Physical Entity, Interaction and Pathway to build a representation of the small molecules, complexes and physical interactions that compose biological pathways and process. Existing ontologies, such as the GO, NCBI taxonomy (**www.ncbi.nlm.nih.gov/Taxonomy/taxonomyhome.html**) and the Cell Type Ontology (**obo.sourceforge.net/cgi-bin/detail.cgi?celltype**), are being used wherever possible by the BIOPAX project. **Kai Runte** described several standards and an ontology relevant to proteomics, which are being developed as part of the HUPO Proteomics Standards Initiative (**psidev.sourceforge.net**). The HUPO effort casts a wide net, aiming to standardize not only the representation of experimental approaches and minimal data requirements — much as the MGED ontology and MIAME do for the microarray community — but also data formats generated and interpreted by instrumentation.

In the ***Functional Genomics Applications*** session, talks covered various aspects of using ontologies in genomic databases and other genomic contexts. Biological databases use ontologies covering several different subdisciplines of biology; **Judith Blake** presented the representative example of the Mouse Genome Informatics databases (Bult *et al.*, 2004), which provides its users a convenient interface to GO, mouse-specific anatomy and phenotype vocabularies, and other controlled vocabularies. **Anastasia Nikolskaya** described the classification of proteins by PIR (Wu *et al.*, 2004) superfamilies, and how that approach complements functional annotation with GO terms. **Karen Eilbeck** presented the Sequence Ontology (SO), which aims to unify the description of sequence features across many sources and many formats. SO covers locatable sequence features (such as *exon, promoter* or *binding_site*) and their properties (sequence attributes such as *maternally_imprinted_gene*, or sequence and chromosomal variations). SO-based annotation is described in detail in the accompanying article (Eilbeck and Lewis, 2004).

In the final session, ***From Resource to Application***, speakers reported on a wide range of applications, most of which involve combining ontologies with other types of data. Two presentations focused on the use of GO in combination with similarity measures: **Antonio Sanfilippo** described weighted links between the three ontologies of GO that can uncover connections between annotated gene products. In **Olivier Bodenreider**'s presentation, semantic similarity between GO terms was used to facilitate gene expression analysis.

The remaining talks all highlighted the application of multiple ontologies to an area of interest. **Matt Mailman** returned to phenotype annotation, from the perspective of using existing ontologies to capture desired information in an object model for storage of phenotypic data being developed at NCBI. **Gilberto Fragoso** reported on the NCI Thesaurus, which addresses the needs of the cancer research community for disease description; also see his review in this issue (Fragoso *et al.*, 2004). **Gloria Despacio-Reyes** discussed the information relevant to crop research and the ontologies used by the International Rice Research Institute. In a summary of terminology relevant to pathology, **Roger Brown** of GlaxoSmithKline provided a perspective from a community that has adopted ontologies recently and needs to adopt and integrate several vocabularies to model experiments and results in sufficient detail to accommodate the needs of regulatory organizations and research scientists. **Mike Waters** described the combination of traditional toxicology and pharmacology with 'omics' scale technologies, and standards and ontologies that are emerging to model toxicogenomics data. The need for standardization in toxicology and pathology, where a plethora of use cases and complexity brought by long established domains such as toxicology meets high-throughput technologies, are explored further by **Sansone** *et al.* in this issue (Sansone *et al.*, 2004).

It is clear that there is a great deal of activity and cooperation between the various disciplines that make up the SOFG community. In particular, the improvements in ontology editors and the applications that use ontologies will continue to bear fruit for the bench biologist, who can expect to use GO annotations when analysing microarray data, retrieve microarray data efficiently from LIMS systems and repositories, and retrieve information efficiently from journal articles

Copyright © 2005 John Wiley & Sons, Ltd.

by using ontologies. That the biologist is often unaware of the gory details of an ontology and its relative complexity indicates that the community is making progress with the technology. Presentations from SOFG2 are available at **www.sofg.org/meetings/sofg2004/index.html**

### Acknowledgements

The authors thank Nancy Place (The Jackson Laboratory) and Jim Wolff (University of Pennsylvania) for conference organization, and Chris Stoeckert, who was volunteered as the SOFG2 host. SOFG2 was funded by the National Human Genome Research Institute, the National Institute for Environmental Health Sciences, the Natural Environment Research Council, GlaxoSmithKline and the MGED Society.

### References

Anatomical Dictionary for the Adult Mouse; **http://www.informatics.jax.org/searches/anatdict_form.shtml**.

BioImage; **www.bioimage.org**.

BIOPAX; **www.biopax.org**.

Brazma A, Hingamp P, Quackenbush J, *et al.* 2001. Minimum information about a microarray experiment (MIAME) — toward standards for microarray data. *Nature Genet* **95**: 365–371.

Bult CJ, Blake JA, Richardson JE, *et al.* The Mouse Genome Database (MGD): integrating biology with the genome *Nucleic Acids Res* **32**: D476–D481.

Cell Type Ontology; **obo.sourceforge.net/cgi-bin/detail.cgi?cell-type**.

ChEBI; **www.ebi.ac.uk/chebi**.

DAG-Edit; **godatabase.org/dev/index.html**.

Eilbeck K, Lewis SE. 2004. Sequence ontology annotation guide. *Comp Funct Genom* (in press).

Feng W, Wang G, Zeeberg BR, *et al.* 2003. Development of gene ontology tool for biological interpretation of genomic and proteomic data. *AMIA Annu Symp Proc* **2003**: 839.

Fragoso G, de Coronado S, Haber M, Hartel F, Wright L. 2004. Overview and utilization of the NCI Thesaurus. *Comp Funct Genom* (in press).

Goble CA, Wroe C. 2004. The Montagues and the Capulets. *Comp Funct Genom* (in press).

Hide W, Smedley D, McCarthy M, Kelso J. 2003. Application of eVOC: controlled vocabularies for unifying gene expression data. *C R Biol* **326**: 1089–1096.

Horrocks I, Patel-Schneider PF, van Harmelen F. 2003. From SHIQ and RDF to OWL: the making of a web ontology language. *J Web Semant* **1**: 7–26.

Kanehisa M, Goto S, Kawashima S, Okuno Y, Hattori M. 2004. The KEGG resource for deciphering the genome. *Nucleic Acids Res* **32**: D277–D280.

Lussier YA, Li J. 2004. Terminological mapping for high throughput comparative biology of phenotypes. *Pac Symp Biocomput* **2004**: 202–213.

McCray AT, Nelson SJ. 1995. The representation of meaning in the 19 UMLS. *Methods Inf Med* **34**: 193–201.

MISFISHIE; **scgap.systemsbiology.net/data/misfishie/**.

NCBI Taxonomy; **www.ncbi.nlm.nih.gov/Taxonomy/taxonomy-home.html/**.

Noy NF, Crubezy M, Fergerson RW, *et al.* 2003. Protégé-2000: an open-source ontology-development and knowledge-acquisition environment. *AMIA Annu Symp Proc* **2003**: 953.

OBO; **obo.sourceforge.net**.

Parkinson HE, Aitken S, Baldock RA, *et al.* 2004. The SOFG Anatomy Entry List (SAEL): an annotation tool for functional genomics data. *Comp Funct Genom* (in press).

PATO; **obo.sourceforge.net/cgi-bin/detail.cgi?poav**.

PSI; **psidev.sourceforge.net/**.

Reactome; **www.reactome.org**.

Rosse C, Mejino JL. 2003. A reference ontology for biomedical informatics: the Foundational Model of Anatomy. *J Biomed Inform* **36**: 478–500.

Sansone SA, Morrison N, Rocca-Serra P, Fostel J. 2004. Standardization initiatives in the (eco)toxicogenomics domain: a review. *Comp Funct Genom* (in press).

SOFG; **www.sofg.org**.

Stevens R, Goble CA, Horrocks I, Bechhofer S. 2002. Building a bioinformatics ontology using OIL. *IEEE Trans Inf Technol Biomed* **6**: 135–141.

Stoeckert C, Parkinson H. 2003. The MGED ontology: a framework for describing functional genomics experiments. *Comp Funct Genom* **4**: 127–132.

Wroe CJ, Stevens R, Goble CA, Ashburner M. 2003. A methodology to migrate the gene ontology to a description logic environment using DAML + OIL. *Pac Symp Biocomput* **2003**: 624–635.

Wu CH, Nikolskaya A, Huang H, *et al.* 2004. PIRSF: family classification system at the Protein Information Resource. *Nucleic Acids Res* **32**: D112–D114.

Zeeberg BR, Feng W, Wang G, *et al.* 2003. GoMiner: a resource for biological interpretation of genomic and proteomic data. *Genome Biol* **4**: R28.

Copyright © 2005 John Wiley & Sons, Ltd. 

## References

[PDF_00405] Brazma A., Hingamp P., Quackenbush J., Sherlock G., Spellman P., Stoeckert C., Aach J., Ansorge W., Ball C. A., Causton H. C. (2001). Minimum information about a microarray experiment (MIAME)-toward standards for microarray data.. Nat Genet.

[PDF_00409] Bult Carol J., Blake Judith A., Richardson Joel E., Kadin James A., Eppig Janan T., Baldarelli R. M., Barsanti K., Baya M., Beal J. S., Boddy W. J. (2004). The Mouse Genome Database (MGD): integrating biology with the genome.. Nucleic Acids Res.

[PDF_00418] Feng Weimin, Wang Geoffrey, Zeeberg Barry R., Guo Kejiao, Fojo Anthony T., Kane David W., Reinhold William C., Lababidi Samir, Weinstein John N., Wang May D. (2003). Development of gene ontology tool for biological interpretation of genomic and proteomic data.. AMIA Annu Symp Proc.

[PDF_00427] Hide Winston, Smedley Damian, McCarthy Mark, Kelso Janet (2003). Application of eVOC: controlled vocabularies for unifying gene expression data.. C R Biol.

[PDF_00433] Kanehisa Minoru, Goto Susumu, Kawashima Shuichi, Okuno Yasushi, Hattori Masahiro (2004). The KEGG resource for deciphering the genome.. Nucleic Acids Res.

[PDF_00436] Lussier Y. A., Li J. (2004). Terminological mapping for high throughput comparative biology of phenotypes.. Pac Symp Biocomput.

[PDF_00439] McCray A. T., Nelson S. J. (1995). The representation of meaning in the UMLS.. Methods Inf Med.

[PDF_00444] Noy Natalya F., Crubezy Monica, Fergerson Ray W., Knublauch Holger, Tu Samson W., Vendetti Jennifer, Musen Mark A. (2003). Protégé-2000: an open-source ontology-development and knowledge-acquisition environment.. AMIA Annu Symp Proc.

[PDF_00454] Rosse Cornelius, Mejino José L. V. (2003). A reference ontology for biomedical informatics: the Foundational Model of Anatomy.. J Biomed Inform.

[PDF_00461] Stevens Robert, Goble Carole, Horrocks Ian, Bechhofer Sean (2002). Building a bioinformatics ontology using OIL.. IEEE Trans Inf Technol Biomed.

[PDF_00464] Stoeckert Christian J., Parkinson Helen (2003). The MGED ontology: a framework for describing functional genomics experiments.. Comp Funct Genomics.

[PDF_00467] Wroe C. J., Stevens R., Goble C. A., Ashburner M. (2003). A methodology to migrate the gene ontology to a description logic environment using DAML+OIL.. Pac Symp Biocomput.

[PDF_00471] Wu Cathy H., Nikolskaya Anastasia, Huang Hongzhan, Yeh Lai-Su L., Natale Darren A., Vinayaka C. R., Hu Zhang-Zhi, Mazumder Raja, Kumar Sandeep, Kourtesis Panagiotis (2004). PIRSF: family classification system at the Protein Information Resource.. Nucleic Acids Res.

